# MADOKA: an ultra-fast approach for large-scale protein structure similarity searching

**DOI:** 10.1186/s12859-019-3235-1

**Published:** 2019-12-24

**Authors:** Lei Deng, Guolun Zhong, Chenzhe Liu, Judong Luo, Hui Liu

**Affiliations:** 1 School of Computer Science and Engineering, Central South University, Changsha, 410075 China; 2 Department of Radiation Oncology, the Affiliated Changzhou No.2 People’s Hospital of Nanjing Medical University, Changzhou, China; 3 Lab of Information Management, Changzhou University, Changzhou, 213164 China

**Keywords:** Protein structure alignment, Structural neighbor searching, Parallel programming

## Abstract

**Background:**

Protein comparative analysis and similarity searches play essential roles in structural bioinformatics. A couple of algorithms for protein structure alignments have been developed in recent years. However, facing the rapid growth of protein structure data, improving overall comparison performance and running efficiency with massive sequences is still challenging.

**Results:**

Here, we propose MADOKA, an ultra-fast approach for massive structural neighbor searching using a novel two-phase algorithm. Initially, we apply a fast alignment between pairwise structures. Then, we employ a score to select pairs with more similarity to carry out a more accurate fragment-based residue-level alignment. MADOKA performs about 6–100 times faster than existing methods, including TM-align and SAL, in massive alignments. Moreover, the quality of structural alignment of MADOKA is better than the existing algorithms in terms of TM-score and number of aligned residues. We also develop a web server to search structural neighbors in PDB database (About 360,000 protein chains in total), as well as additional features such as 3D structure alignment visualization. The MADOKA web server is freely available at: http://madoka.denglab.org/

**Conclusions:**

MADOKA is an efficient approach to search for protein structure similarity. In addition, we provide a parallel implementation of MADOKA which exploits massive power of multi-core CPUs.

## Background

Protein structure alignment can reveal remote evolutionary relationships for a given set of proteins, and thus helps significantly to understand the function of proteins [1–7]. In the last two decades, numerous computational tools have been proposed to perform optimal protein structure alignment such as DALI [8], CE [9], SAL [10], FATCAT [11], TM-align [12], Fr-TM-align [13], FAST [14], CASSERT [15], DeepAlign [16], MICAN-SQ [6], etc. Because of the complexity of protein structures, these methods are mainly different from presentations of structures and similarity scoring matrices. In practice, most structure alignment approaches begin with constructing a set of equivalent residues [13]. The structural similarity score is then calculated using various steps and metrics, and a dynamic programming procedure is employed to acquire the final result. A bottom-up scheme by assembling small alignment fragments to build a global alignment is brought in many methods [8, 13, 17–19]. This involves iterative comparisons and merges of many fragments, and its computational tasks become very heavy when making all-against-all operations [20].

Among structural alignment algorithms, root-mean-square deviation (RMSD), is the most widely used metric between a pair of length-equal structures for performance assessment, which is defined as:

1 $$ RMSD=\sqrt{\frac{1}{N}\sum_{i=1}^{N}d_{i}^{2}}  $$

where *N* is the number of aligned pairs of residues, and *d*_*i*_ is the distance between the *i*^*th*^ pair of residues. However, as Zhang [21] and Skolnick [22] figured out, a small number of local structural deviations may result in a large RMSD value, even the global topologies of the compared structures are very similar. Additionally, the RMSD of randomly chosen structures depends on the lengths of compared structures. TM-score [21] has overcome these deficiencies, which is a more accurate measure in evaluating the alignment quality of full-length pairwise protein structures, and it is independent of protein lengths:

2 $$ TM-score=Max\left [ \frac{1}{L}\sum_{i=1}^{N_{ali}}\frac{1}{1+\left (\frac{d_{i}}{d_{0}} \right)^{2}} \right ]  $$

here, *L* denotes the length of the original structure, *N*_*ali*_ is the number of aligned residue pairs, and $d_{0}=1.24\sqrt [3]{L-15}-1.8$.

Protein structure similarity searching is a one-against-all structure alignment process, which is especially important in situations where sequence similarity searches (e.g., BLAST [23]) fail or deliver too few clues. Large-scale structural similarity searches using traditional structure alignment algorithms is typically time-consuming [24, 25]. A number of approaches have been proposed to accelerate the speed of structure similarities searching, such as [26], CASSERT [24] and ppsAlign [25]. Despite significant advances in structure alignment algorithms, protein structure similarity search against a large structural database is still a great challenge, as protein structures are highly complex and protein 3D structure repositories are becoming increasingly huge, such as Protein Data Bank (PDB) [27].

In this paper, we describe a new method named MADOKA for fast and accurate protein structure similarity searching. MADOKA is designed to filter out the structures with low secondary structural similarity in the first phase as initial alignment, and perform precise alignment in the second phase as accurate alignment. MADOKA also benefits from highly parallelized programming by using multi-core processors to accelerate processes of protein structure similarity neighbor search.

## Results

SCOP and CATH [28] are used as standards for assessing the structure alignment in various methods. However, proteins that differ from fold families in the SCOP and CATH categories may contain significant structural similarity [13]. We have geometric measure benchmarks purely to evaluate the structure alignment quality between pairwise proteins.

### Datasets

We use three datasets to assess the performance of MADOKA. The first dataset TM-align is obtained from the TM-align paper [12], which includes 200 non-homologous protein structures from PDB ranging in size from 46 to 1058 residues. We get (200×199)/2=19,900 protein pairs in total. The second dataset comes from MALIDUP [29], which contains 241 manually curated pairwise structure alignments homologous domains originated from internal duplication. The third is MALISAM [30], which consists of 130 protein pairs that are different in terms of SCOP [31] folds but structurally analogous.

MADOKA employs the secondary structure elements and the backbone C *α* coordinates of the protein structures for alignment.

### Performance comparison with existing structure alignment techniques

**Fig. 1:**
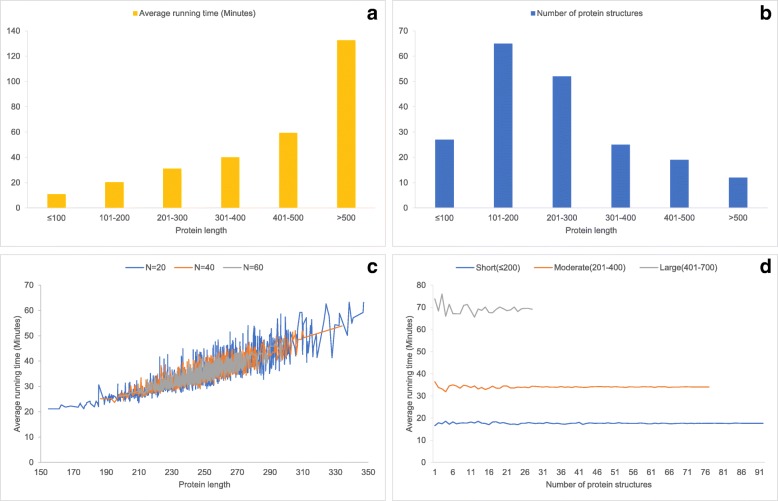
Computing time and amount of protein at different protein sizes. **a** shows the average computing time of proteins in the TM-align dataset for structural similarity searching against the whole PDB database by using MADOKA. **b** indicates the number of structures at different protein sizes in the TM-align dataset. **c** shows average running time curves with respect to randomly selected proteins from entire TM-align dataset (N=20, 40 and 60 is the number of selected proteins each time). **d** shows average running time corresponding to three different group of protein split by lengths

We have performed comparison experiments on a workstation computer with two Intel Xeon E5-2630 v3 processors and 64GB of memory. The result of the alignments generated by MADOKA and CE [9], SAL [10], TM-align [12], Fr-TM-align [13] on the TM-align dataset is shown in Table 1. MADOKA achieves the best performance in the RMSD and TM-score metrics. Most importantly, the speed of MADOKA is much faster than the other four algorithms and its total time consumption was about 265 seconds, indicating the filtering process and parallel computing play a key role in improving search speed. By the first-phase alignment, our method largely narrows down the number of pairwise proteins for precise alignments to be done in the second phase, 11,052 pairs complete both phases in total, which account for about 55.5% of all 19,900 structure pairs. Moreover, the implementation of MADOKA is a concurrent system [32] that runs many alignments for different pairs on different CPU cores at the same time. We use MADOKA to search structure neighbors against the entire PDB database for each protein in the TM-align dataset. The calculation time corresponding to proteins with different lengths is shown in Fig. 1a, and the distribution of protein number with respect to the protein length is shown in Fig. 1b. We can see that the larger the size of protein structure, the longer the calculation time is needed. Most proteins are in 100aa-300aa in length, and the number of protein longer than 500aa is small. It is worth noting that the total calculation time depends on the number of proteins, but the average calculation time is only related to the size of protein structure. Moreover, we carry out another experiment to check the relationship between average calculation time and size of protein structure. From the entire dataset, we randomly extract certain number of proteins (N=20, 40 and 60) to execute searching task, and then compute the average protein length and average running time. This process is repeated for 1000 times. The result is shown in Fig. 1c, we find that three curves of average running time overlap to each other, meanwhile increase gradually with the protein length. This result indicates that the average running time is largely affected by the size of protein structure, not the number of proteins. Finally, we split the proteins into three groups by their length, i.e. short (≤200aa), moderate (201aa-400aa) and large (401aa-700aa). For each group, we randomly extract increasing number of proteins to execute the searching task, and the average running time is computed. The process is repeated till every protein in a group is selected at least one time. As shown in Fig. 1d, the curves of average running time regarding to each group keep steady, while they differ largely from each other for different size of protein structures.

**Table 1:** Alignment performance comparison on the TM-align dataset

Method	RMSD	TM-score	Running Time (s)
CE	6.30	0.273	0.52
SAL	6.96	0.320	2.47
TM-align	4.99	0.348	0.13
Fr-TM-align	4.73	0.365	1.65
MADOKA	4.07	0.562	0.02

Next, we conduct performance evaluation on the other two datasets for MADOKA and five different methods, including DeepAlign [33], DALI [8], MATT [34], Formatt [35] and TM-align [12] in Table 2. *N*_*ali*_ measure represents the total count of aligned residues in each pairwise structure alignment [36]. In this test, we skip the first phase in order to verify the validity of the second phase. Among these approaches, MADOKA obtains highest TM-score and number of aligned residues (*N*_*ali*_). Moreover, MADOKA’s calculation time is far less than other methods.

**Table 2:** Performance of six pairwise structure alignment tools on benchmarks MALIDUP and MALISAM

Benchmark	Method	Nali	RMSD	TM-score	Total Time (s)
MALIDUP	DeepAlign ^∗^	85.5	2.61	0.622	10.2
	DALI ^∗^	83.5	2.65	0.600	115.3
	MATT ^∗^	82.3	2.47	0.608	63.0
	Formatt ^∗^	70.6	2.19	0.542	85.1
	TM-align ^∗^	87.0	2.62	0.631	6.4
	MADOKA	91.7	3.43	0.631	1.2
MALISAM	DeepAlign ^∗^	61.3	2.96	0.521	4.3
	DALI ^∗^	61.0	3.11	0.515	47.4
	MATT ^∗^	56.2	2.74	0.486	16.2
	Formatt ^∗^	44.9	2.42	0.411	33.1
	TM-align ^∗^	61.1	3.06	0.517	2.9
	MADOKA	62.8	2.72	0.555	0.7

^*^These are detailed in [33]

### Parameters selection

Note that the LCS length for strings of secondary structural elements of each protein pair will be compared with a threshold. If the length is less than the threshold, the pair will be filtered out. So the threshold for the second phase should be selected properly. The higher the threshold, the fewer pairs will get residue-level alignments. The lower the threshold, the weaker the acceleration effect of the first phase. The length of LCS for pairwise strings depends mainly on the length of the shorter one. For trade-off between time efficiency and alignment accuracy, we take the threshold as *min*(*m*,*n*)×0.7 in all of our tests. A protein pair will pass the first phase if the LCS length *S*[*m*,*n*]>*threshold*.

Within the TM-align dataset, we choose the gap open penalty of the second phase of MADOKA as 3×10^−6^. For MALIDUP and MALISAM datasets, we specify the gap penalty as 3×10^−6^ and 0.08, respectively. We find that maybe a low gap penalty contributes to a better result for dataset contains many remote homologous protein structures, but it likely just opposite for structurally analogous proteins.

### Case study

**Fig. 2:**
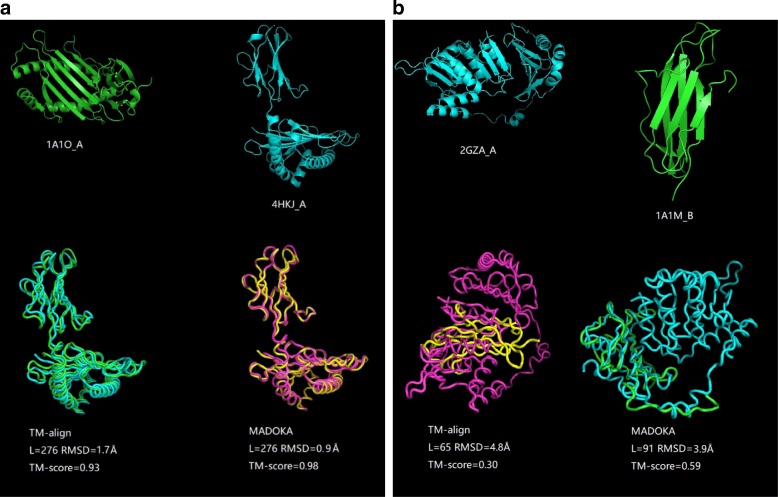
Two examples showing the structural alignments from TM-align and MADOKA. **a** shows alignment between 1A1O_A (276 residues) and 4HKJ_A (277 residues). **b** shows alignment between 2GZA_A (334 residues) and 1A1M_B (99 residues)

As shown in Fig. 2a and b, there are two illustrative examples of TM-align alignments and MADOKA alignments. Benefits from optimal-position based fragment alignment, MADOKA could gain some improvements. Figure 2a shows structural alignments between 1A1O_A and 4HKJ_A, MADOKA is able to align nearly all regions and get a better superposition result, as well as RMSD and TM-score. Figure 2b shows alignments between 2GZA_A and 1A1M_B, MADOKA acquires an optimized aligned position which has a common region of *β*-strands, which obtains higher TM-score and lower RMSD value.

### Web interface

**Fig. 3:**
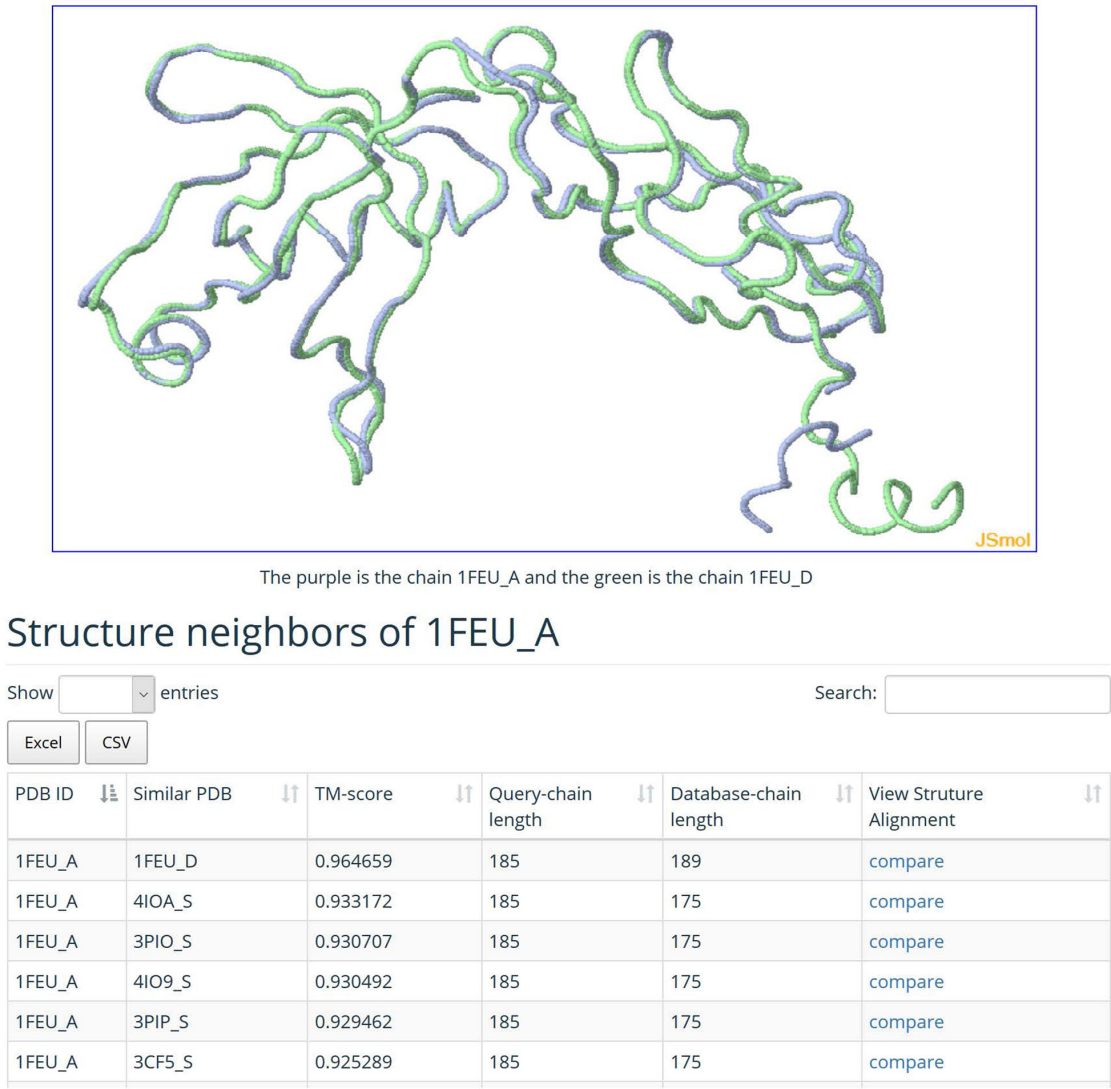
Web page for 3D visualization of structure alignment and structure neighbors

Our MADOKA web server accepts a protein 3D structure file in PDB format or a PDB code as input. MADOKA will check the validity of the input protein, and then conduct structure similarity searching against the whole PDB database. The time required for similarity searching is dependent on the size of the query protein. Most searches can be completed within half an hour. The output consists of a list of structural neighbors, their RMSD and TM-scores for each submitted query protein, which can be downloaded in text format. A unique feature is the 3D visualization of structure alignment for the query protein and its structural neighbors (Fig. 3).

## Discussion

Commonly, there are two kinds of protein structure alignment approaches. The first compares a pair of structures with an a priori specified equivalence between pairs of residues (often offered by sequence or threading alignments [37]). The second is to compare structures under a set of equivalent residues, which is not a priori given; this is an NP-hard problem with no exact solution for an optimal alignment [38]. Accurate protein structure alignment could be complicated and computationally expensive as protein structures are very large and databases are becoming increasingly huge such as PDB. In this study, we integrate SSE and residue similarity to search protein structural similarity neighbors effectively. Moreover, our algorithm focused on searching an optimal aligned position between a short structure and a long structure to obtain a local alignment, rather than an all-to-all residues comparisons based global alignment. The local alignment contributes to higher TM-score, lower RMSD and more aligned residues. A limitation of MADOKA is that it requires specified gap penalty value for residue-level alignment, which may limit its application. However, with the classification of protein data becomes clearer, we believe MADOKA can be a useful fast tool for accurately searching protein structural neighbor in large-scale context.

## Conclusion

In this paper, we proposed a two-phase algorithm, referred to as MADOKA, for protein structural alignment and similarity neighbor searching, together with a web server. The secondary structure element, residue alignment and filtering mechanism are introduced to accelerate the alignment process and performs faster when a parallel implementation is used. Compared to existing representative protein structure alignment methods, MADOKA outperforms about 6, 20 and 100 times faster than TM-align, CE and SAL on large-scale datasets, respectively. Meanwhile, MADOKA achieves better alignment quality than a couple of methods. We expect MADOKA to be applied to structure-based protein interaction and function predictions [39–42].

## Methods

### Overview of MADOKA

**Fig. 4:**
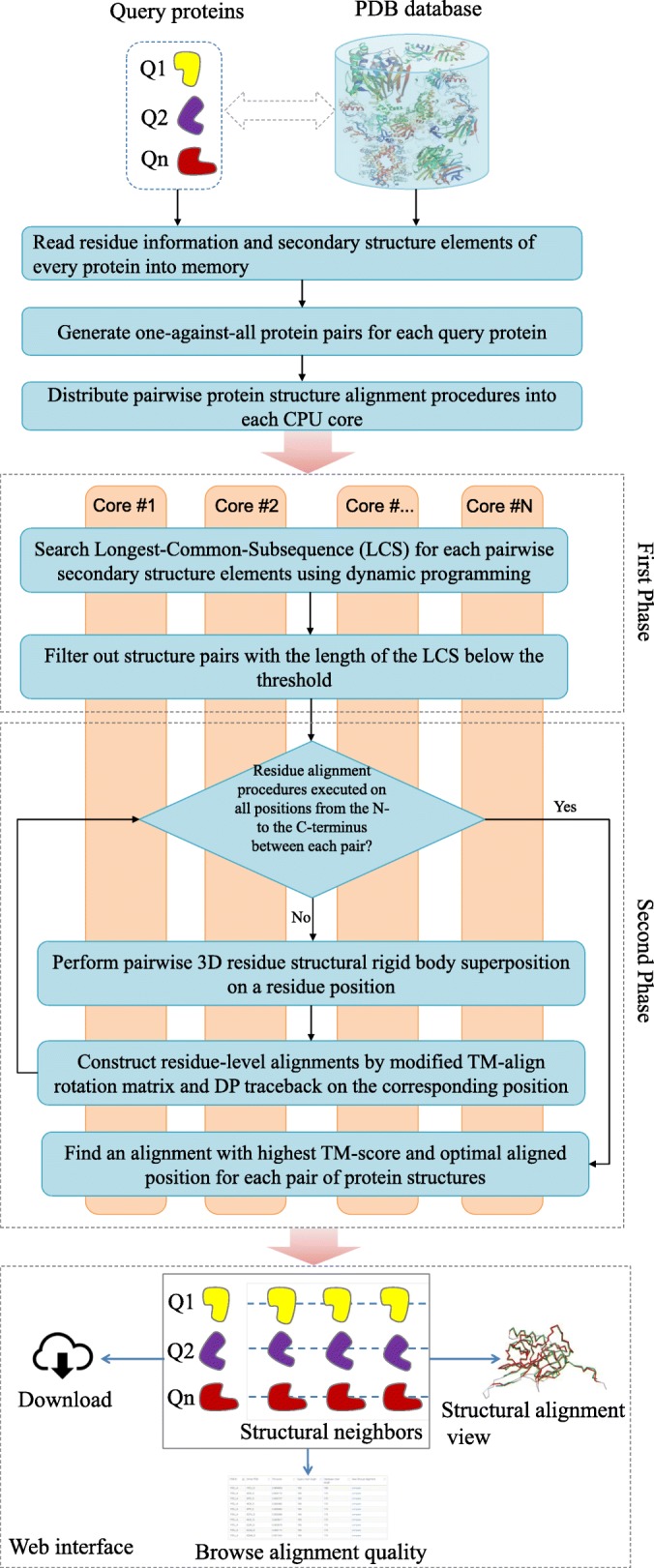
Schematic diagram of MADOKA algorithm and the web interface. The algorithm involves two steps: 1) Search for Longest-Common-Subsequence (LCS) for each pairwise secondary structure elements using dynamic programming, and then structure pairs with the length of the LCS below the threshold are removed; 2) Pairwise 3D residue structural rigid body superposition is performed and residue-level alignments are constructed, and the best alignment with the highest TM-score and optimally aligned position for each pair of protein structures is selected

MADOKA performs one-against-all structure alignment procedure between the query protein and all proteins in the database. The illustrative workflow is shown in Fig. 4. The algorithm of MADOKA is composed of two phases. In the first phase, we represent pairwise protein structures as two strings of secondary structure units, and then conduct initial alignment between the secondary structure sequences by marking the Longest-Common-Subsequence (LCS) by dynamic programming. In the second phase, for each pairwise proteins with initial alignment score larger than a predefined threshold, we run pairwise 3D residue structural alignments by rigid body superposition and modified TM-align rotation matrix to pick up an alignment with highest TM-score (the comprehensive description of these two phases are showed in the following two sections). For versatility, The MADOKA implementation is written in C++ standard syntax and standard concurrency library, and thus supports multiple compilers natively and can run on many operating systems such as Microsoft Windows, macOS and Linux without modification. The program will decide whether to use multi-threading mode depending on the scale of input pairs; if the program is in parallel state, there will be multiple simultaneous executions of the algorithms for different pairs. The MADOKA website is developed using Perl, JavaScript, jQuery(AJAX) and CSS, and calls the MADOKA program for protein structure similarity searching.

### First phase: initial alignment

First of all, we denote pairwise protein structures A and B as two strings of secondary structure types (*α*-helix, *β*-strand, coil and others) by using the DSSP [43] program, each character in a string corresponds with the secondary structure element (SSE) of a residue:

3 $$ A=[SSE_{1}^{A},SSE_{2}^{A},...,SSE_{m}^{A} ]  $$

4 $$ B=[SSE_{1}^{B},SSE_{2}^{B},...,SSE_{n}^{B}]  $$

*m*, *n* is the number of residues in protein structures *A* and *B*, respectively.

The initial alignment is obtained by marking the two strings using the Longest-Common-Subsequence(LCS) between them, the effective solution using dynamic programming of the LCS problem is given in Eq. (5).

5 $$ S[\!i,j]\,=\,\left\{\begin{array}{ll} 0 & \text{} i=0\;or\;j=0\\ S[i-1,j-1]+1 & \text{} i,j>0\;and\;A_{i}=B_{j}\\ max(S[i-1,j],S[i,j-1]) & \text{} i,j>0\;and\;A_{i}\neq B_{j}\\ \end{array}\right.  $$

**Fig. 5:**
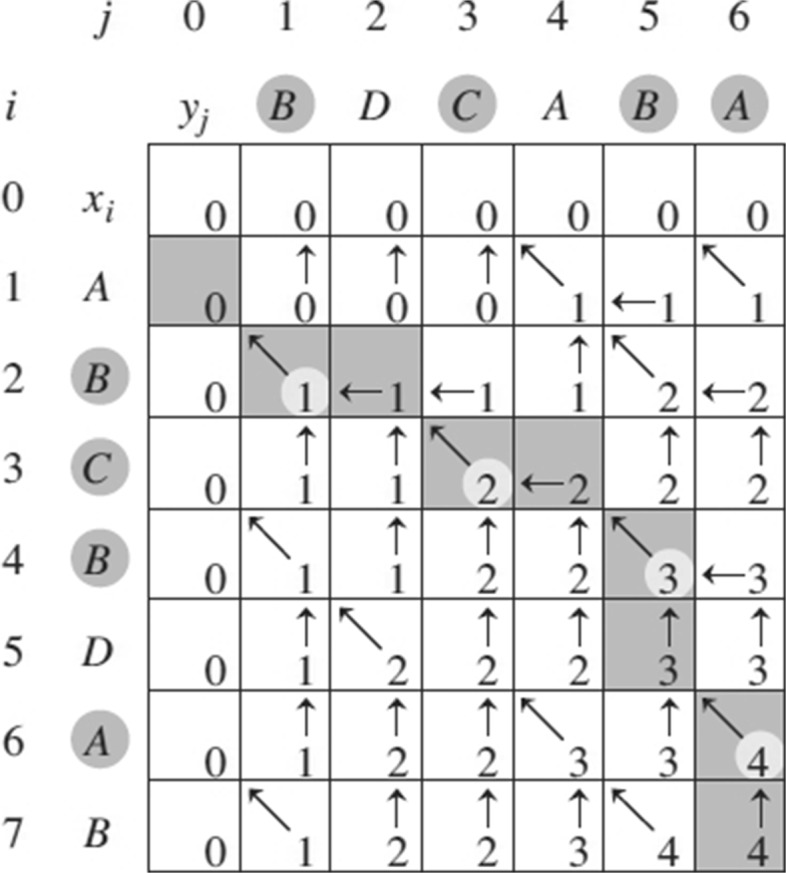
Scoring matrix for LCS problem and dynamic programming backtrack


In which *S* is a (*m*+1)×(*n*+1) dimension scoring matrix, *S*[*i*,*j*] is the length of LCS ranging from *A*_1_ to *A*_*i*_ and *B*_1_ to *B*_*j*_. Then, *S*[*m*,*n*] is the length of LCS for the global *A* and *B*. Finally, make a traceback on *S* to get an optimal path for initial alignment. An example is demonstrated in Fig. 5 [44], and the detailed step of backtracking for constructing initial alignment is described in Algorithm 1.

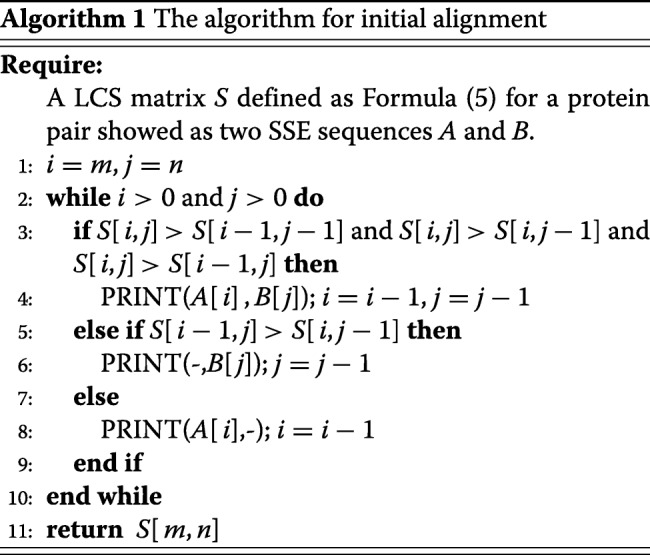



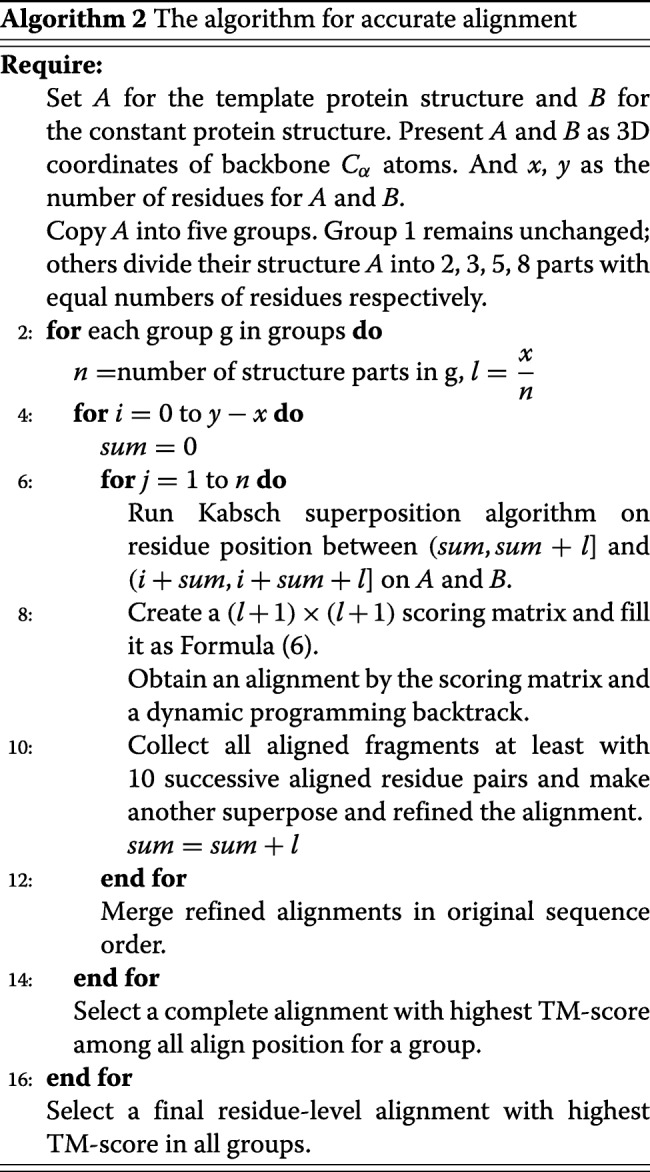


### Second phase: accurate alignment

We set a threshold for each pairwise alignment in the first phase. Structures passed the first phase are being further aligned to obtain the accurate alignment in the second phase. This phase employs the 3D coordinates of backbone *C*_*α*_ atoms for a pair of aligned protein structures A and B. We pick the short structure that contains fewer residues as template structure, and the other as constant structure. Then we set *L*_*t*_ as the length of the template and *L*_*c*_ as the length of the constant. Starting with the template structure, we superpose it to the corresponding residues of the constant residues according to Kabsch algorithm [45]. Secondly, we create a new scoring matrix for template and a fragment of the same length with template on constant. The matrix is defined as:

6 $$ M[i,j]=\left\{\begin{array}{l} 0 \;\;when\;i=0\;or\;j=0 \\ Max \left\{\begin{array}{l} M[i-1,j]+g\\ M[i,j-1]+g\\ M[i-1,j-1]+\frac{1}{1+d_{ij}^{2}/d_{0}(L_{min})^{2}}\\ \end{array}\right. \end{array}\right.  $$

where *g* is the gap penalty customized by user, *d*_*ij*_ is the distance of the *i*^*th*^ residue in template structure and the *j*^*th*^ residue in constant structure under the superposition, and $d_{0}(L_{min}) =1.24\sqrt [3]{L_{min}-15}-1.8$ which *L*_*min*_ being the length of the template. The formula above is a modified TM-align rotation matrix [12] definition. An alignment can be constructed by a dynamic programming backtrack on M. An alignment consists of residue pairs which are aligned or a gap inserted between a pair. Next, we collect all fragments on the alignment with at least 10 successive aligned residue pairs, then superpose this set of fragments onto the constant structure again. A new alignment is generated by another traceback with a new matrix. Then we perform a gapless threading which is composed of all (*L*_*c*_−*L*_*t*_+1) iterations with residue location shifting from the N- to the C-terminus between the template and constant. Next, we choose an alignment with maximum TM-score computed by the superposed template and the corresponding fragment on the constant structure. Be aware that the optimal alignment is between the whole template and the fragment which has an optimized position on the constant and the same number of residues as the template.

There is usually a strong relationship between the converged superposition and the length of superposed fragment, so we create five groups; each group contains a copy of the template structure. Then each group divides their template into several parts; all parts in a group have an equal length. The number of parts in each group is 1, 2, 3, 5, and 8, respectively. Next, we take parts in each group to have the superposed, DP and gapless threading procedures with the order of template residue sequence, and then combine all sub-structure alignments into a complete alignment for each group. Eventually, the alignment with the highest TM-score among all number of parts is selected as the final accurate alignment. A more concrete description for the algorithm of phase two is presented in Algorithm 2.

## Data Availability

The web server, experiment benchmarks and the source code of the standalone program of MADOKA are freely available at http://madoka.denglab.org/.

## Abbreviations

3D: 3-Dimensional
aa: Amino acid
BLAST: Basic local alignment search tool
CASSERT: *C*_*α*_ atom (C), angle defined by vectors between successive *C*_*α*_ atoms (A), secondary structure element (SSE), residue type (RT)
CATH: Protein class (C), architecture (A), topology (T) and homologous superfamily (H)
CE: Combinatorial extension
CPU: Central processing unit
DALI: Distance mAtrix aLIgnment
DP: Dynamic programming
DSSP: Define secondary structure of proteins
FATCAT: Flexible structure alignment by chaining AFPs (Aligned Fragment Pairs) with Twists
FAST: A recursive acronym of FAST alignment and search tool
GB: Gigabyte
LCS: Longest-common-subsequence
MALIDUP: Manual alignments of duplicated domains
MALISAM: Manual alignments of structurally analogous motifs
MATT: Multiple alignment with translations and twists
MICAN-SQ: Multiple-chain complexes, inverse direction of secondary structures, *C*_*α*_ only models, alternative alignments, non-sequential alignments, and sequential alignment
NP-hard: Non-deterministic polynomial-time hard
PDB: Protein data bank
ppsAlign: Parallel protein structure alignment
RMSD: Root-mean-square deviation
SAL: Structure alignment
SCOP: Structural classification of proteins
TM-score: Template modeling score
SSE: Secondary structure element
